# Prevalence of musculoskeletal symptoms from online learning during the COVID-19 epidemic: a systematic review and meta-analysis

**DOI:** 10.12688/f1000research.152382.1

**Published:** 2024-07-11

**Authors:** Tanawat Gotum, Orawan Keeratisiroj, Wutthichai Jariya

**Affiliations:** 1 Faculty of Public Health, Naresuan University, Tha Pho, Phitsanulok, 65000, Thailand

**Keywords:** COVID-19, Meta-analysis, Musculoskeletal pain, Online learning, Student, Systematic review

## Abstract

**Purpose:**

The objective of this research was to assess the prevalence of musculoskeletal symptoms in online students.

**Materials and methods:**

A systematic review and meta-analysis were performed by searching the PubMed, Cochrane Library, SCOPUS, Web of Science, ScienceDirect, ProQuest, CINAHL plus with full text, and Wiley InterScience databases. A total of 3,749 studies were identified between January 2020 and December 2023. The Joanna Briggs Tool for studies reporting prevalence was used to assess the quality of studies. Jamovi 2.4 was used in the meta-analysis.

**Results:**

Sixteen studies were included and used for the meta-analysis. There were 6 studies of high quality, 9 studies of medium quality and 1 study of low quality. The areas with the highest prevalence of musculoskeletal pain were the neck (51%, 95% CI = 36–66%), lower back (51%, 95% CI = 42–59%) and shoulder (36%, 95% CI = 26–47%).

**Conclusions:**

The shift to online learning during the COVID-19 pandemic has emerged as a potential factor influencing musculoskeletal pain in students. Educational institutions should study the duration of online learning that begins to impact student injury outcomes.

## Introduction

The coronavirus disease 2019 (COVID-19) pandemic has emerged as a global health crisis due to its widespread transmission and ease of airborne contagion. With more than 703 million confirmed cases and 774 million fatalities worldwide as of December 31, 2023, ^1^ countries around the globe have implemented stringent measures to curb the spread of the virus. Among these measures, city lockdowns have emerged as a prominent strategy to contain the COVID-19 pandemic. However, these lockdowns have significantly impacted the daily lives of individuals worldwide. ^2^ City lockdowns or lockdowns restrict public movement, border crossings, and dine-in services at restaurants, fundamentally altering people’s daily routines globally. The impact extends to both work and education, with working professionals transitioning to remote work arrangements and students shifting to online learning platforms. ^3^

As the COVID-19 pandemic continues to spread unabated across many countries, including Thailand, educational institutions have adopted online learning strategies to mitigate the risk of infection. ^4^ Online learning has significantly impacted students’ daily lives, as they engage in virtual instruction via electronic devices connected to the internet. Instead of traditional classroom settings, students spend time in front of computers, smartphones, or tablets, typically for 6-8 hours per day, 3-5 days per week. This shift to remote learning has led to a substantial decrease in physical activity among students. A study by the Thai Center for Knowledge Development on Physical Activity revealed that even in normal circumstances, Thai children engage in sedentary behavior for more than 13 hours per day. However, the COVID-19 pandemic has exacerbated this issue, with sedentary behavior increasing to 14 hours per day for Thai children. This sedentary lifestyle is largely attributed to the shift toward online learning and prolonged screen time. ^5^ The World Health Organization recommends that children engage in at least 60 minutes of physical activity daily. ^6^ Data collected in Thailand over the past decade indicate that only 26% of Thai children meet these physical activity guidelines. Notably, during the COVID-19 pandemic, since 2020, the proportion of Thai children meeting the recommended physical activity standards has further declined to 17%. ^5^ International studies have also documented a decline in physical activity among university students and younger people during the COVID-19 pandemic. A study in Indonesia revealed that university students’ physical activity levels decreased to an average of 60.92 minutes per week during the pandemic. ^7^ Similarly, a German study involving 1,711 children and adolescents revealed a reduction in sports participation among those aged 4-17 years during the pandemic. ^8^ Furthermore, an Italian study revealed a decrease in physical activity among university students during the pandemic, with lower levels of physical activity associated with an increased risk of musculoskeletal pain onset and worsening pain symptoms. ^9^

Prolonged physical inactivity or maintaining a static posture for extended periods can lead to musculoskeletal problems, including fatigue, localized pain, and other symptoms. ^10^ This issue extends beyond students and affects teachers as well. Research investigating musculoskeletal disorders (MSDs) among students engaged in online learning during the COVID-19 pandemic has revealed an increased prevalence of muscle aches and pains among this population. A study involving a sample of 261 students who transitioned to online learning during the pandemic revealed that 80% of participants reported experiencing headaches, eye strain, and neck pain following online classes. ^11^ A study in Iran compared musculoskeletal symptoms among 220 faculty members at Yazd University before and during the COVID-19 pandemic using the Standardized Nordic Questionnaire. The findings revealed a statistically significant increase in musculoskeletal complaints among university faculty during the pandemic. ^12^ MSDs are a prevalent global health concern. A study in the United States involving 654 university students reported a 12.5% prevalence of joint disorders among participants. ^13^ Another study among computer-using university students reported that 23% of respondents had used medication to manage musculoskeletal symptoms. ^14^ A South African study involving 145 participants revealed a high prevalence of musculoskeletal pain (89.7%) among university students. ^15^ Additionally, a Chinese study among first-year university students revealed a significant association between prolonged internet use and musculoskeletal pain. ^16^ These findings collectively demonstrate the increased prevalence of MSDs among online learners across various countries.

Musculoskeletal injuries can be classified into two main categories: acute and cumulative. Acute injuries result from a direct impact or force, such as a blow or crush, while cumulative injuries develop gradually over time due to repetitive or prolonged postures or activities. ^17^ Online learning, for instance, can contribute to cumulative musculoskeletal injuries. A study in Jordan examining neck pain among online learners using smartphones revealed that 43.9% of students who spent 10-30% of their day engaged in online learning reported neck pain. ^18^ Additionally, a study investigating the causes of musculoskeletal symptoms during lockdown measures revealed that among 319 participants aged 18-60 years who were confined to their homes, the most common reasons for pain were increased phone usage (43.7%), prolonged sitting (41.3%), and lack of physical activity (29.4%). The most prevalent pain locations were the lower back (62.2%), neck (48%), and upper back (35.4%). ^19^ MSDs can cause pain in muscles, tendons, bones, or joints throughout the body and can significantly impact daily life if severe. These disorders can affect individuals of all ages, including children, not just those in the working population. ^20^

MSDs have been extensively studied in various occupations, including athletes (e.g., runners, ^21^ ballet dancers ^22^ and healthcare workers (e.g., dentists, ^23^ nurses ^24^). However, a systematic review and meta-analysis of MSD prevalence among online learners during the COVID-19 pandemic has not yet been conducted. Therefore, this study aimed to systematically review and meta-analyze the prevalence of MSDs among online learners during the COVID-19 pandemic to quantify the magnitude of the problem and inform future research directions.

## Methods

### Protocol and registration

This study is reported following the NEW Preferred Reporting Items for Systematic reviews and Meta-Analyses (PRISMA) guidelines. ^25^ The study protocol was registered in the International Prospective Register of Systematic Reviews (PROSPERO) (Registration number: CRD42022335229).

### Search strategies

The search for relevant studies was conducted in electronic databases, including Medline via PubMed, Cochrane Library, Wiley InterScience, SCOPUS, ProQuest, and SciendDirect. The search included studies published between January 2020 and December 2023. Keywords were searched individually and in combination using Boolean operators such as “OR” and “AND.” The following medical subject headings (MeSH) were used: Prevalence, Musculoskeletal, Low back pain, School student, College student, University student, Adolescent, Online Learning, Distance Education, Distance Learning, Online Education, Remote Learning, COVID-19, Computer, and Smartphone (Extended data: Supplementary file 1).

### The eligibility criteria

Research articles meeting the following criteria were included in the study: 1) target population: university students; 2) study context: online learning during the COVID-19 pandemic; 3) research objective: to investigate the prevalence of MSDs among online learners; 4) study design: cross-sectional study; 5) publication date: January 2020 to December 2023; 6) language: English; and 7) publication type: peer-reviewed journal.

Exclusion criteria: 1) Nonreporting of prevalence: Research articles that did not report the prevalence of MSDs among online learners were excluded. 2) Inaccessible full-text: Research articles for which the full text could not be accessed were excluded.

### Study Selection and Data Extraction

Two independent reviewers (TG and OK) screened the search results, initially assessing potential studies based on titles and abstracts. For studies that appeared to meet the inclusion criteria, the full-text articles were retrieved. Any discrepancies in the screening reports were resolved through consensus with a third reviewer (WJ).

Data were extracted from articles that met the inclusion and exclusion criteria for further analysis. The data extracted included author, year, country, sampling frame, study sample size, response rate, period of data collection, tool for MSDs, period for pain, prevalence of symptoms, and duration of use/online.

### Quality Assessment of Included Studies

The Joanna Briggs Institute (JBI) critical appraisal checklist for prevalence studies was used to evaluate the methodological quality of the included studies. ^26^ This tool assesses studies based on nine questions. A score of 1 is awarded for each “yes” answer, while a score of 0 is given for “no,” “unclear,” or “not applicable” responses. Overall quality scores ≤ 4, 5-7, and ≥ 8 were considered low, moderate, and high, respectively. Two reviewers (TG and OK) independently conducted the quality assessments, and any discrepancies were resolved through discussion.

### Data Analysis

Jamovi 2.4 was used in the meta-analysis. Prevalence estimates for MSDs were calculated using percentages and 95% confidence intervals (CIs). Heterogeneity of the included studies was assessed using Cochran’s Q test with a significance level of 0.05. If there was significant heterogeneity between studies ( *p* value < 0.05), a random effects model was used to estimate the pooled prevalence. For studies with nonsignificant heterogeneity ( *p* value ≥ 0.05), a fixed-effects model was used to calculate the pooled prevalence.

Publication bias was evaluated using funnel plots, Egger’s test, with a significance level of 0.05. If publication bias was detected, efforts were made to identify and report potential sources of bias.

## Results

### Study selection and characteristics

**Figure 1.**
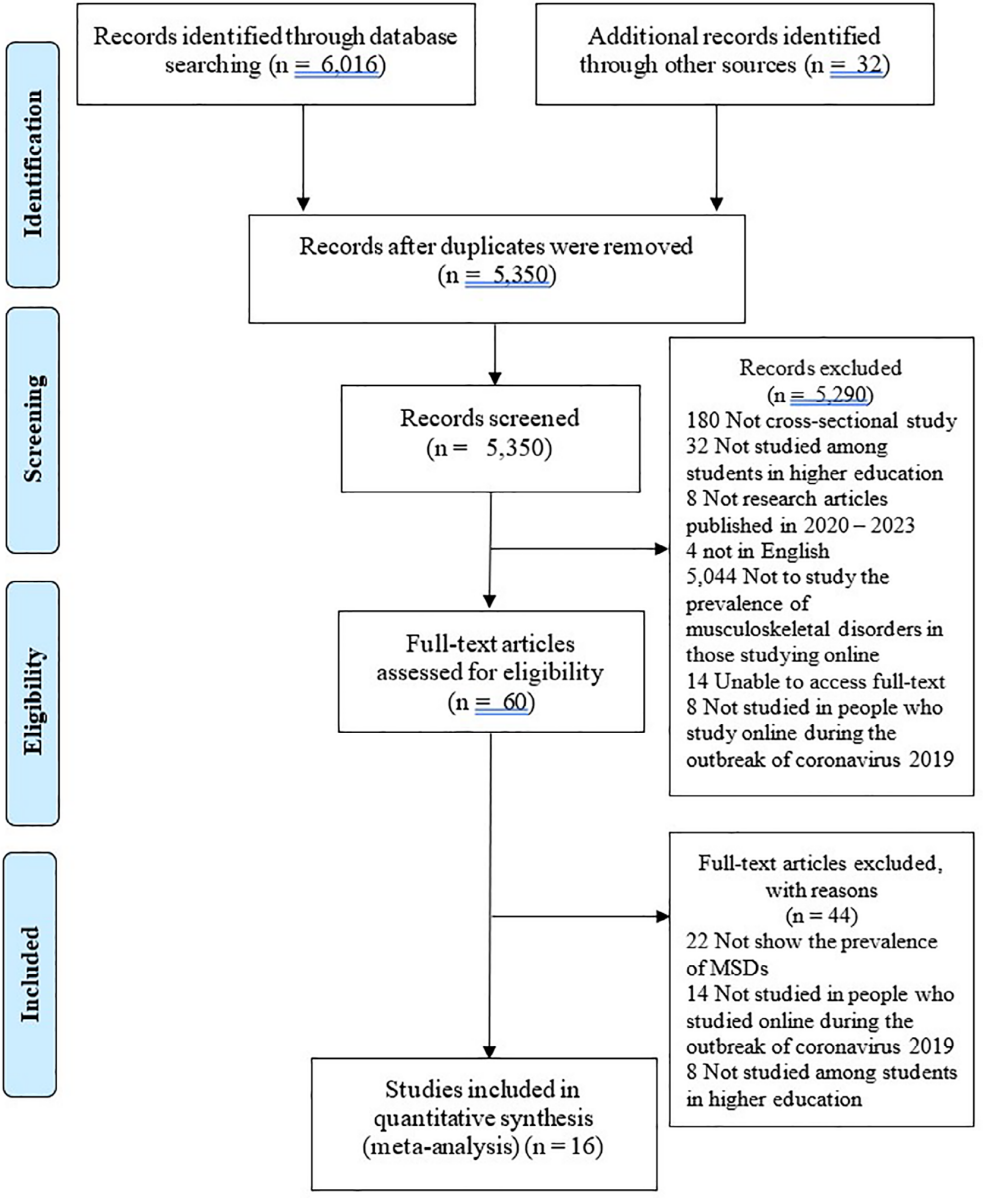
The PRISMA flow chart of the study selection process. ^25^

The search yielded 6,016 articles and an additional 32 articles from the reference checking of the collected studies. After checking for duplicates and applying the inclusion and exclusion criteria, 5,350 articles remained. Based on the title and abstract, 60 articles remained. After a thorough review of the full-text articles, 16 articles remained. These were entered into the article assessment and meta-analysis ( Figure 1). ^25^

As shown in Table 1, the characteristics of the study population were as follows: medical students (n = 5), ^27^^–^^31^ nursing students (n = 3), ^31^^–^^33^ dental students (n = 1), ^29^ pharmacy students (n = 1), ^29^ physical therapy students (n = 1), ^34^ physical education and sports students (n = 1), ^35^ and university students without specified faculty (n = 6). ^32^^,^^36^^–^^40^ The sample sizes of these studies ranged from 120 to 3,705 participants. The mean age was 22 years (Extended data: Supplementary file 2).

**Table 1.** Study characteristics.

No.	Author	Year	country	Period of data collection	Tool for MSD	Period for pain	Sampling frame	Sample size	Age (yrs)	Respons rate	Duration of use/online
1	Elghomati	2022	Turkish Republic of Northern Cyprus	1 and 22October 2020	Cornell musculoskeletal discomfort questionnaire	1 week	The Eastern Mediterranean University (EMU)	544	24.6 (range 17-33)	90.67%	Smartphone 1-2 hr 45(8.2%) 3-4 hr 151 (27.7%) 5-6 hr 141 (25.9) >6 hr 177 (32.5)
2	Gomes	2021	Brazil	December 2020	Standardized Nordic Musculoskeletal Questionnaire	1 week	Medical student	154	22 (Range 18-34)	32%	>4 hours = 86 (55.8%); bettween 2 and 4 hours = 39 (25.3%); <2 hours = 29 (18.9%)
3	Karingada	2022	India	15 July and 10 August 2020	Self-reported MSD (Not standard tool)	3 months	Undergraduate students in India	261	19.32 ± 2.37 (Range 20-25)	37.28%	
4	Leirós-Rodríguez	2020	spain	16 March and 11 May, 2020	Standardized Kuorinka Modified Nordic Questionnaire	7 Days, 12 months & during lockdown months	Two Spanish universities (Universidad de León and Universidad de Valladolid	1,198	22.8 ± 5.9	3.8%	
5	Roggio	2021	Italy	8 February to 21 March 2021	Not standard tool ( https://forms.gle/dzCy8MSdYUdq3wEb8)	4/9/12 months	Italian university students	1,654	22.51 ± 3.12	80.92%	
6	Salameh	2022	Jordan	July to September 2021	Not standart tool		The study targeted undergraduate medical students	282	18-22	100%	< 6 hr (49/282) > 6 hr (233/282)
7	Silișteanu	2022	Romania	2021 for a period of 5 months	Not standard tool	during online education	The College of Physical Education and Sports of the University of Suceava	218	18-40	52.91%	
8	Sirajudeen	2022	Saudi Arabia	March and May 2021	Standardized Nordic musculoskeletal questionnaire	12 months	University students in the Kingdom of Saudi Arabia during the COVID-19 pandemic	313	22.6 ±4.08 (Ranged 18–45)	82.37%	About an hour 2 (0.6%) 1–3 h 22 (7%) 3–5 h 51 (16.3%) 5–7 h 75 (24%) 7 h or more 163 (52.1%)
9	Nermen	2022	Saudi Arabia	April 2022 to July 2022	1. Vernon Mior’s Neck Disability Index (NDI) 2. Roland–Morris Disability Questionnaire (for LBP)		Nursing students	120	21.4 ± 1.7	100%	Mean of 9.1 (±4.6 SD) study hours per week 87.5% of participants use digital devices every day
10	Magdalena Janc	2023	Poland	July 2020 and October 2020 before the COVID-19 pandemic and during the period from October 2020 to June 2021	The Nordic Musculoskeletal Questionnaire		The study was student status at one of the three universities	914	21.7 ± 2.2	100%	
11	Direksunthorn	2023	Thai	April to June 2022	The Nordic Musculoskeletal Questionnaire		Nursing students	3,705	17–25	80.23%	
12	Ferlito 1	2023	Italy	30 August 2021, and concluding on 4 October 2021	Modified questionnaire	1-6 months 12 months more than 12 months	Study in physiotherapy at the universities of Catania, Messina, and Palermo	201	between 22 and 25	100%	Spending 15–22 hr per week distance learning, followed by 7–14 hr for 27.4%, 23–30 hr for 10.4%, and a small minority (4.5%) who spent more than 30 hr
13	Almhdawi	2021	Jordan	2020 to June 2021	Neck Disability Index (NDI)		Medical, dentistry, pharmacy, and nursing students at multiple Jordanian universities	485	20.6 ± 2.0	100%	
14	Samaraha	2022	Jordan	May and August 2021	Modified Nordic Musculoskeletal questionnaire	1 week and 12 months	Two Jordanian universities’ medical students	593	20.89 ± 2.13	100%	
15	Harithasan	2022	Malaysia		The Standardized Nordic Questionnaire	1 week	Undergraduate students	179	18-25	100%	
16	El-Bidawy	2021	Saudi Arabia		The self administered structured online questionnaire consisted of 25 questions	6 weeks	Medical students	188	18-30	100%	

### Study quality

**Figure 2.**
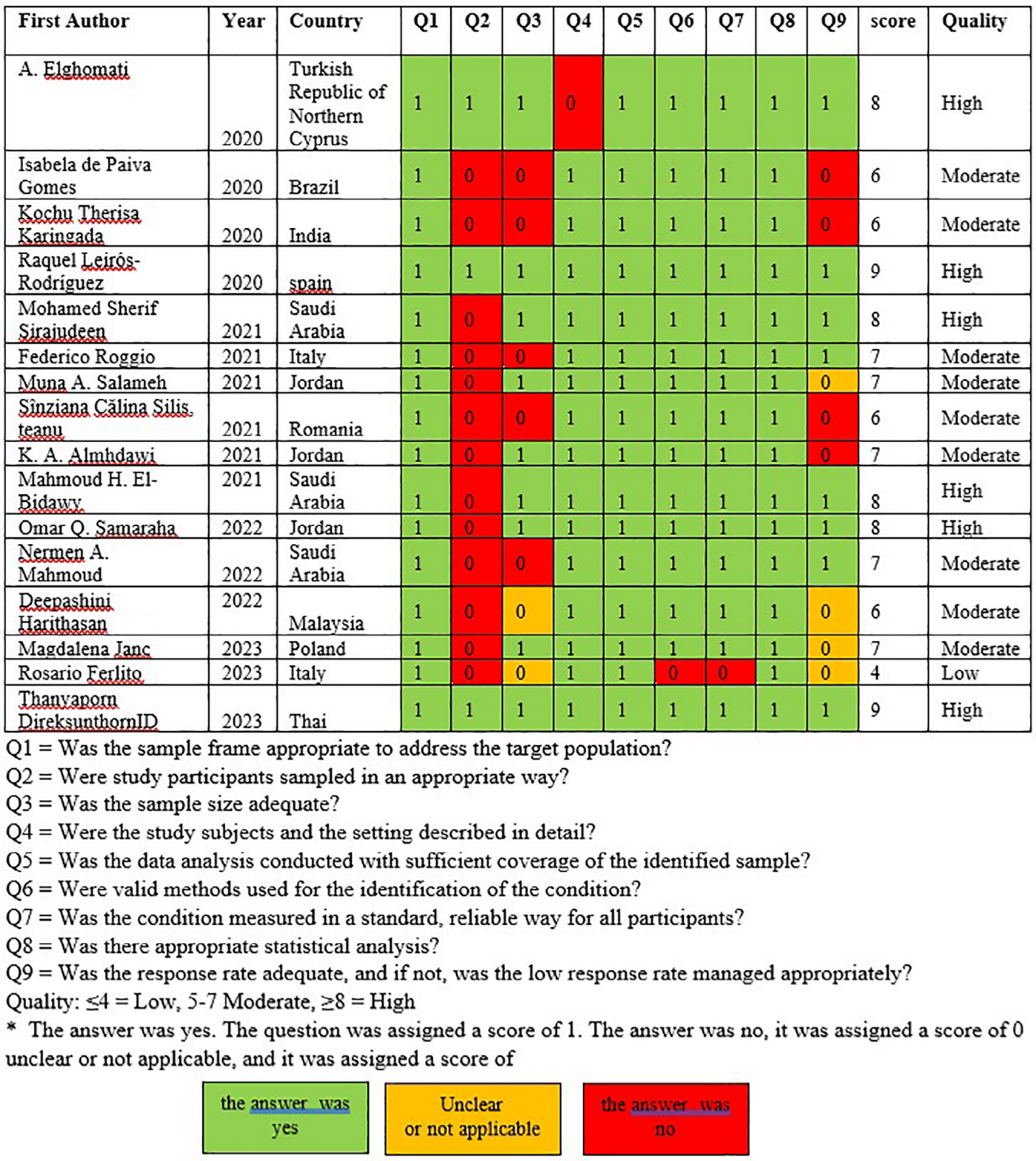
Assessment of study quality.

Assessment of the research quality by two researchers (TG and OK) revealed that there were six high-quality studies, nine moderate-quality studies, and one low-quality study. The studies investigating musculoskeletal symptoms were conducted in six European countries, ^34^^–^^36^^,^^38^^,^^39^^,^^41^ nine Asian countries, ^29^^–^^33^^,^^37^^,^^40^^,^^42^ and one South American country ( Figure 2). ^27^

### Meta-analysis

A meta-analysis was conducted to determine the prevalence of musculoskeletal symptoms in 9 body regions according to the Standardized Nordic Questionnaire (SNQ). ^43^ Additional body regions were added to align with the prevalence reported in the reviewed articles, including the upper arm and lower arm, for a total of 11 regions. The results are presented for three groups: 1) axial body: neck, upper back, and lower back; 2) upper limbs: shoulder, upper arm, lower arm, elbow, and wrist/hand; and 3) lower limbs: hip/thigh, knee, and ankle/foot.

**Figure 3.**
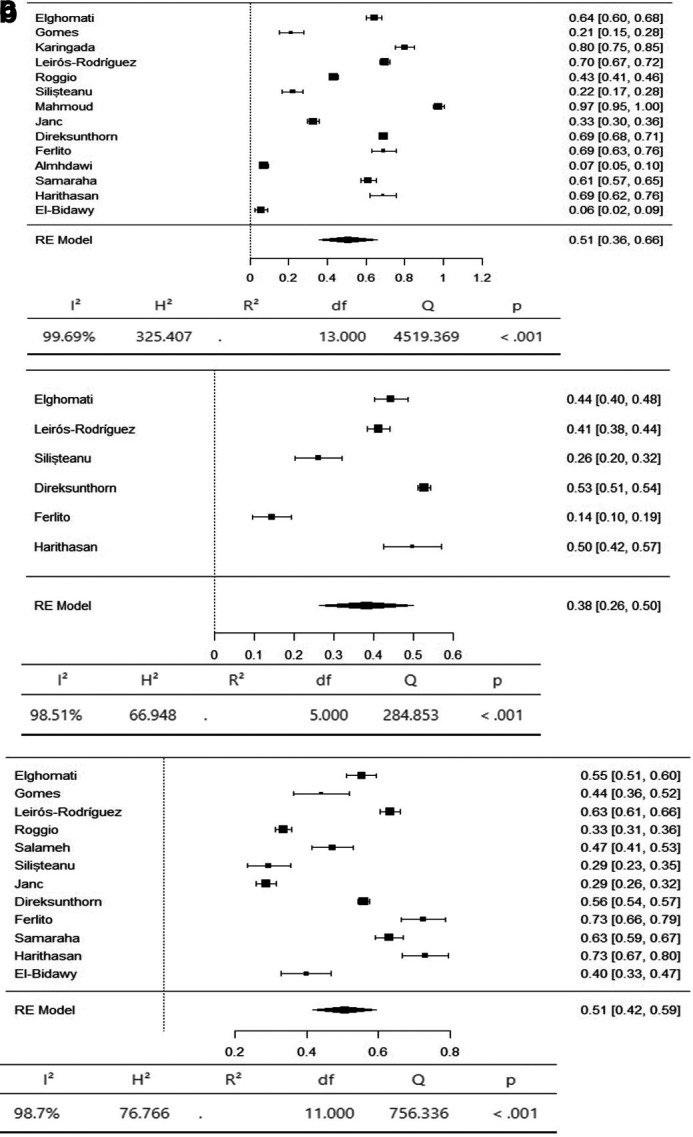
Forest plots displaying the meta-analysis for the prevalence of symptoms in the axial body.

**Figure 4.**
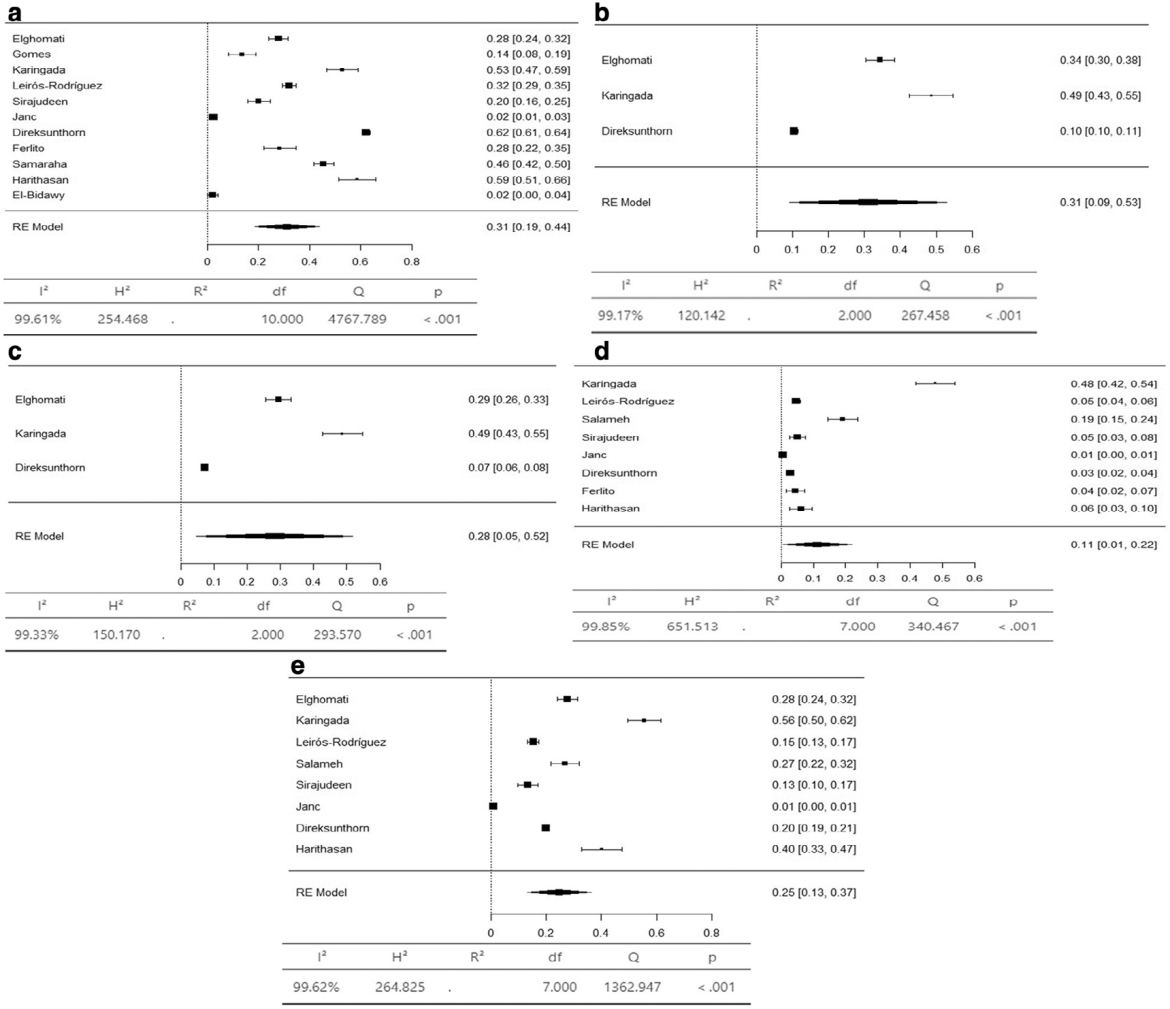
Forest plots displaying the meta-analysis for the prevalence of symptoms in the upper limbs.

**Figure 5.**
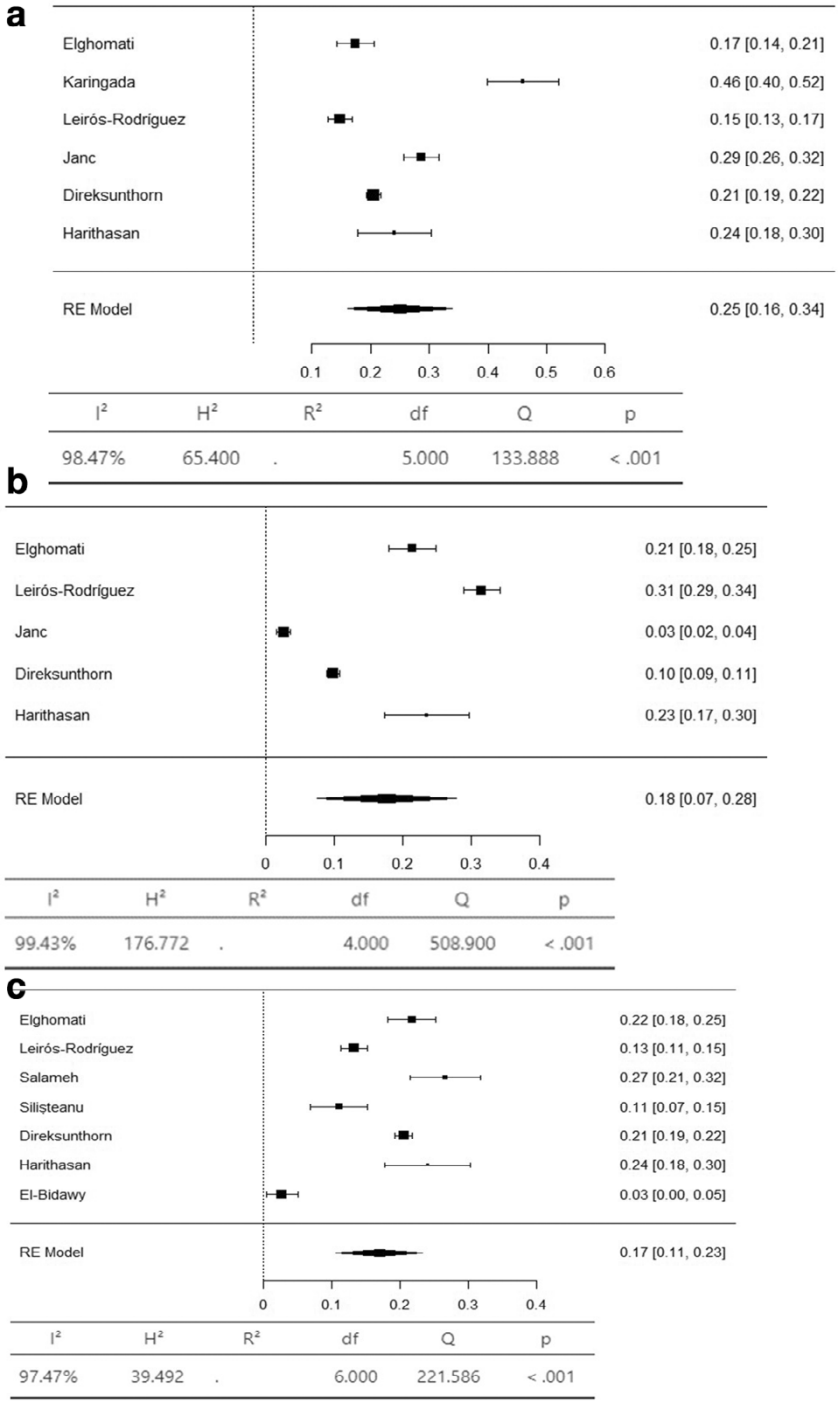
Forest plots displaying the meta-analysis for the prevalence of symptoms in the lower limbs.

The axial body region had the highest prevalence of musculoskeletal symptoms, with the neck (95% CI 36-66%) and lower back (95% CI 42-59%) having an equal prevalence of 51%. The prevalence of hypertension in the upper back was 36% (95% CI 26–47%) ( Figure 3). The upper limbs had the highest prevalence of musculoskeletal symptoms, with the shoulder (95% CI 19-44%) and upper arm (95% CI 9-53%) having an equal prevalence of 31%. The lower arm had a prevalence of 28% (95% CI 5-52%). The elbow had the lowest prevalence of musculoskeletal symptoms, at 11% (95% CI 1 - 22%) ( Figure 4). The lower limbs had the highest prevalence of injuries, with the hip having a prevalence of 25% (95% CI 16–34%). The prevalence of knee involvement was 18% (95% CI 7-28%), and the prevalence of ankle/foot involvement was 17% (95% CI 11-23%) ( Figure 5).

Subgroup analyses were conducted for the axial body (neck, upper back, lower back) to examine the heterogeneity between studies. The heterogeneity was not significant for any of the subgroups (I ^2^ = 99.69-98.20). Egger’s regression test was used to assess publication bias. No evidence of publication bias was found in the meta-analysis of the overall prevalence of pain ( *p* value < 0.001). Similarly, no evidence of publication bias was found for the upper limbs (shoulder, upper arm, elbow, forearm, wrist/hand) (I ^2^ = 99.85-99.17, *p* value < 0.001) or lower limbs (hip, knee, lower leg) (I ^2^ = 99.43-97.47, *p* value < 0.001) (Extended data: Supplementary file 3 and Table 2).

**Table 2.** The results for all 11 body regions.

Anatomical site	Number of studies	Sample size	I ^2^	Tau ^2^	Effect size (95%CI)	*p* value
Neck	14	10,414	99.69	0.0818	0.36-0.66%	<0.001
Upper back	6	6,958	98.20	0.0168	0.26-0.47%	<0.001
Lower back	12	9,830	99.69	0.0818	0.49-0.51%	<0.001
Shoulders	11	8,250	99.61	0.0447	0.19-0.44%	<0.001
Upper arms	3	4,510	99.17	0.0368	0.09-0.53%	<0.001
Elbows	8	7,053	99.85	0.0236	0.0.1-0.22%	<0.001
Lower arm	3	4,510	99.33	0.0428	0.05-0.52%	<0.001
Wrists/Hands	8	7,396	99.62	0.0279	0.13-0.37%	<0.001
Hips/Thighs	6	6,801	98.47	0.0118	0.16-0.34%	<0.001
Knees	5	6,540	99.43	0.0132	0.07-0.28%	<0.001
Ankles/Foots	7	6,314	97.47	0.0068	0.11-0.23%	<0.001

## Discussion

This systematic review and meta-analysis aimed to investigate the prevalence of musculoskeletal symptoms in different body regions among individuals engaged in online learning during the COVID-19 pandemic. A total of 16 studies were included, comprising six high-quality studies, nine moderate-quality studies, and one low-quality study. The studies were conducted in six European countries, nine Asian countries, and one South American country. The sample sizes ranged from 120 to 3,705 participants.

The results of this meta-analysis on the prevalence of musculoskeletal symptoms among individuals engaged in online learning during the COVID-19 pandemic revealed that the most frequently reported pain locations were the neck, lower back, and shoulders. The least frequently reported pain locations were the upper arm, lower arm, and knee. These findings are consistent with previous studies on musculoskeletal injuries among work-from-home employees during the COVID-19 pandemic, which reported prevalence rates of 20.3-76.9% for neck pain, 19.5-74.1% for lower back pain, and 3.0-72.9% for shoulder pain. ^44^ Additionally, a study conducted among students and faculty members of the Faculty of Public Health in Thailand reported a 34% prevalence of upper arm pain during periods of online learning and work from home, ^45^ which is similar to the findings of our meta-analysis.

For the lower limbs, the results of this study revealed that the most prevalent musculoskeletal symptom was located in the hip, with an overall prevalence of 25% [95% confidence interval (CI) 16–34%]. This finding is consistent with a study conducted among online teachers during the COVID-19 pandemic in Brazil, which reported a prevalence of hip pain of 25%. ^46^ This is likely due to prolonged sitting in the same position during online learning, which can lead to more hip pain than in other areas, such as the knee and ankle/foot, which are more commonly associated with pain in athletes. ^47^

Musculoskeletal pain in students can arise from various factors, including poor posture, prolonged sitting or standing, improper ergonomics, ^48^ stress, ^40^ and lack of physical activity. ^9^ Studies investigating online learning environments suggest a potential association between prolonged screen time and poor posture. ^28^^,^^49^ This finding aligns with established research demonstrating that maintaining a hunched posture during prolonged reading, texting, or laptop use can strain muscles and lead to pain in the neck, back, and shoulders. ^39^^,^^50^^,^^51^ Notably, the current study did not examine the specific risk factors associated with musculoskeletal pain in this online learning population.

Studies conducted prior to the COVID-19 outbreak have established the prevalence of musculoskeletal pain. ^52^ However, the COVID-19 pandemic appears to have significantly increased its prevalence. Reports indicate an increase in the prevalence of musculoskeletal pain, ranging from 15.60% to 64.40%, possibly attributable to increased screen time and reduced physical activity levels associated with lockdowns. ^53^ While respiratory complications are well established in patients with COVID-19, musculoskeletal manifestations are increasingly being recognized. ^54^^,^^55^ During the acute phase, patients with COVID-19 may experience fatigue, myalgia, arthralgia, back pain, and chest pain. These symptoms can also persist after contracting COVID-19. ^56^ The specific causes of musculoskeletal involvement in patients with COVID-19, potentially including viral effects, medications, and immobilization, remain under investigation. ^57^^,^^58^ Emerging evidence suggests an increased prevalence of musculoskeletal symptoms during COVID-19 infection.

This systematic review has some limitations that should be considered. First, the causes of musculoskeletal pain resulting from online learning have not yet been adequately studied, as insufficient and diverse research is available. However, there is a clear understanding of the causes of MSDs from online learning, which are primarily related to physical ergonomic factors. ^59^ Second, the study designs included in this review were cross-sectional, which means that the duration of online learning that leads to musculoskeletal pain could not be analyzed. Finally, this study could not analyze ergonomic factors to identify the types of devices involved. Therefore, further research is needed to investigate the duration of online learning that contributes to musculoskeletal pain. This information could be used to develop guidelines for dividing online learning into appropriate periods throughout the day to minimize musculoskeletal injuries among students. Additionally, further research is needed to examine the types of electronic devices that have the greatest impact on musculoskeletal injuries. This information could be used to inform recommendations for device selection and usage to reduce the risk of musculoskeletal injuries among students.

## Conclusion

The shift to online learning is another factor that may affect musculoskeletal pain in students. However, there is insufficient evidence to determine the specific duration of online learning per day that begins to impact the presence of musculoskeletal injuries in learners.

### Implications for Rehabilitation

• Setting up an ergonomic workspace at home (chair height, monitor position, etc.) and maintaining good posture while using electronic devices.
• Encourage teachers to incorporate short physical activity breaks into online lessons. This could involve simple stretches, standing desks, or short bursts of jumping jacks.
• Develop resources or guidelines for students on recognizing early signs of discomfort and appropriate self-care strategies (e.g., applying heat/ice, modifying postures, etc.).

## Author contributions

TG, OK and WJ designed the study. TG and OK acquired the data. TG and OK analyzed and interpreted the data. TG and OK wrote the initial draft. All authors critically reviewed and approved the final manuscript. All authors had final responsibility for the decision to submit for publication.

## Data Availability

No data are associated with this article. Mendeley Data: Musculoskeletal symptoms from online learning: systematic review and meta-analysis, https://data.mendeley.com/datasets/jhggs6tbw4/2. ^60^ This project contains the following underlying data: Supplementary file 1: Search strategies. Supplementary file 2: Study prevalence. Supplementary file 3: Funnel plot. PRISMA_2020_checklist Data are available under the terms of the Creative Commons Attribution 4.0 International license (CC-BY 4.0).
