# Supplementary material for: Prevalence of musculoskeletal symptoms from online learning during the COVID-19 epidemic: a systematic review and meta-analysis
Source: F1000Res. 2025 Jun 5;13:790. Originally published 2024 Jul 11. [Version 3] doi: 10.12688/f1000research.152382.3 (PMC12150206; doi:10.12688/f1000research.152382.3)
Supplement: Peer review history

# Reviewer report: Reviewer response for version 3

**DOI:** 10.5256/f1000research.183283.r389977

**Published:** 2025-06-25

**Authors:** Turgay Altunalan

**Affiliations:** 1 Department of Physical Therapy and Rehabilitation, Karadeniz Technical University, Trabzon, Turkey

This version of systematic review is presented in a more comprehensive manner and includes parameters such as pain intensity and location.

Are the rationale for, and objectives of, the Systematic Review clearly stated?

Partly

Is the statistical analysis and its interpretation appropriate?

I cannot comment. A qualified statistician is required.

If this is a Living Systematic Review, is the ‘living’ method appropriate and is the search schedule clearly defined and justified? (‘Living Systematic Review’ or a variation of this term should be included in the title.)

Not applicable

Are sufficient details of the methods and analysis provided to allow replication by others?

Yes

Are the conclusions drawn adequately supported by the results presented in the review?

Partly

Reviewer Expertise:

Physiotherapy

I confirm that I have read this submission and believe that I have an appropriate level of expertise to confirm that it is of an acceptable scientific standard.

# Reviewer report: Reviewer response for version 2

**DOI:** 10.5256/f1000research.180429.r378739

**Published:** 2025-05-24

**Authors:** Turgay Altunalan

**Affiliations:** 1 Department of Physical Therapy and Rehabilitation, Karadeniz Technical University, Trabzon, Turkey

I am pleased to have had the opportunity to evaluate this interesting work.

 This manuscript presents a systematic review and meta-analysis investigating the prevalence and characteristics of musculoskeletal symptoms (MSS) associated with online learning during the COVID-19 pandemic. The authors synthesized data from 16 studies to determine the most commonly affected anatomical regions and potential contributing factors among student populations. Neck, back, and shoulder pain were among the most frequently reported complaints.

1. Title and Abstract is concise and appropriately reflects the content of the article.

The abstract should include more detailed numerical data relevant to meta-analysis reporting, such as the total sample size, number of included studies, effect sizes, and heterogeneity statistics (e.g., p value, I²). These additions would strengthen the clarity and value of the abstract.

1. Introduction effectively contextualizes the shift to online learning during the pandemic and its potential impact on postural health.

However, the authors should more explicitly articulate the knowledge gap that this review aims to address. In addition, relevant and recent literature addressing the postural and ergonomic consequences of prolonged screen use and sedentary behavior should be incorporated to enhance the scholarly depth of the background section.

 3. Discussion

 The study included only studies that gave the frequency of pain in the meta-analysis. However, a comparative analysis should be presented, with studies investigating pain intensity in the discussion section. Are the body parts with the most frequently reported pain and those with the most severe? Is the severity of pain in the body parts where pain is reported also clinically significant?  Comparative comments with the following article results may add value regarding the clinical significance of the article results. https://doi.org/10.1002/ca.24163  

- The discussion appropriately compares findings with existing literature, but the clinical relevance of the results—particularly with ergonomic interventions, physical activity, and health education—should be more thoroughly explored.
- Implications for public health and educational policy should be explicitly stated.
- The study limitations are addressed; however, the fact that all included studies are cross-sectional should be emphasized earlier in the discussion, as this restricts causal inferences.

Are the rationale for, and objectives of, the Systematic Review clearly stated?

Partly

Is the statistical analysis and its interpretation appropriate?

I cannot comment. A qualified statistician is required.

If this is a Living Systematic Review, is the ‘living’ method appropriate and is the search schedule clearly defined and justified? (‘Living Systematic Review’ or a variation of this term should be included in the title.)

Not applicable

Are sufficient details of the methods and analysis provided to allow replication by others?

Yes

Are the conclusions drawn adequately supported by the results presented in the review?

Partly

Reviewer Expertise:

Physiotherapy

I confirm that I have read this submission and believe that I have an appropriate level of expertise to confirm that it is of an acceptable scientific standard, however I have significant reservations, as outlined above.

## References

[1] : Musculoskeletal pain intensity and perceptions during distance learning: A cross-sectional study. *Clin Anat* .2024;37(5) : 10.1002/ca.24163 578-586 10.1002/ca.24163 38546148

# Reviewer report: Reviewer response for version 2

**DOI:** 10.5256/f1000research.180429.r377748

**Published:** 2025-05-08

**Authors:** Mati Pääsuke

**Affiliations:** 1 University of Tartu, Tartu, Estonia

The authors responded satisfactorily to the comments made.The design of this manuscript was improved during the review process.I now recommend that this article be accepted for indexing.

Are the rationale for, and objectives of, the Systematic Review clearly stated?

Yes

Is the statistical analysis and its interpretation appropriate?

Yes

If this is a Living Systematic Review, is the ‘living’ method appropriate and is the search schedule clearly defined and justified? (‘Living Systematic Review’ or a variation of this term should be included in the title.)

Yes

Are sufficient details of the methods and analysis provided to allow replication by others?

Yes

Are the conclusions drawn adequately supported by the results presented in the review?

Yes

Reviewer Expertise:

musculoskeletal disorders

I confirm that I have read this submission and believe that I have an appropriate level of expertise to confirm that it is of an acceptable scientific standard.

# Reviewer report: Reviewer response for version 1

**DOI:** 10.5256/f1000research.167132.r331325

**Published:** 2024-10-31

**Authors:** Mati Pääsuke

**Affiliations:** 1 University of Tartu, Tartu, Estonia

**General Comments**

 This is an interesting systematic review and meta-analysis, assessing the prevalence of musculoskeletal disorders (MSDs) in different body regions among online learners during the COVID-19 pandemic. A total 16 full-text articles from different electronic databases were included in this meta-analysis. The participants were University students with mean age of 22 years from 6 European countries, 9 Asian countries and 1 South American country. This meta-analysis suggested, that the areas with the highest prevalence of MSDs in online learners were the neck (51%), lower back (51%) and shoulder (36%). The Authors concluded that the shift to online learning during the COVID-19 pandemic has emerged as a potential factor influencing musculoskeletal pain in students.

 The manuscript is generally well written. However, the design of this paper should be slightly improved before getting approved for indexing.

1. In my opinion, it is obligatory to present in Abstract and Results (Study selection and characteristics) information about age range and sex (% female) of the participants for better understanding the measured subjects.
2. This study has several additional limiting factors:
  (1) Cultural (participants from 16 countries, and from Europe, Asia and South America) differences may have possibly influenced the individuals’ understanding and interpretation of the musculoskeletal pain.
  (2) The gender-related differences in MSDs  of the participants was not analyzed in this meta-analyses.
  (3) Habitual physical activity of the participants was not analyzed in this study. All these limitations should be presented at the end of the Discussion.

In conclusion, I recommend to accept this manuscript for indexing in bibliographic databases given that the authors address all the minor corrections.

**Specific Comments**

*Abstract*

 Page 1. Please add the information about age range and sex (% female) of the participants.

 Results

 Page 5, Study selection characteristics. Please add information about age range and sex (%female) of the participants.

 Discussion

 Page 12. Please add more limiting factors (see General Comments)

Are the rationale for, and objectives of, the Systematic Review clearly stated?

Yes

Is the statistical analysis and its interpretation appropriate?

Yes

If this is a Living Systematic Review, is the ‘living’ method appropriate and is the search schedule clearly defined and justified? (‘Living Systematic Review’ or a variation of this term should be included in the title.)

Yes

Are sufficient details of the methods and analysis provided to allow replication by others?

Yes

Are the conclusions drawn adequately supported by the results presented in the review?

Yes

Reviewer Expertise:

musculoskeletal disorders

I confirm that I have read this submission and believe that I have an appropriate level of expertise to confirm that it is of an acceptable scientific standard, however I have significant reservations, as outlined above.

# Response

**Published:** 2025-04-08

**Authors:** Orawan Keeratisiroj

**Affiliations:** Facilty of Public Health, Naresuan University, Tha Pho, Phitsanulok, Thailand

**General Comments**

 This is an interesting systematic review and meta-analysis, assessing the prevalence of musculoskeletal disorders (MSDs) in different body regions among online learners during the COVID-19 pandemic. A total 16 full-text articles from different electronic databases were included in this meta-analysis. The participants were University students with mean age of 22 years from 6 European countries, 9 Asian countries and 1 South American country. This meta-analysis suggested, that the areas with the highest prevalence of MSDs in online learners were the neck (51%), lower back (51%), and shoulder (36%). The Authors concluded that the shift to online learning during the COVID-19 pandemic has emerged as a potential factor influencing musculoskeletal pain in students.

 The manuscript is generally well written. However, the design of this paper should be slightly improved before getting approved for indexing.

 In my opinion, it is obligatory to present in Abstract and Results (Study selection and characteristics) information about age range and sex (% female) of the participants for better understanding the measured subjects.

**Reply: **Thank you for your valuable suggestion. We have now included the age range of the participants (17–45 years) in both the Abstract and the Study Selection and Characteristics sections to enhance the readers’ understanding of the studied population. However, we were unable to report the sex distribution (% female) of the participants due to the limitations of the secondary data. Specifically, the primary studies included in this systematic review did not consistently report participants' sex, which precluded us from providing this information.

**This study has several additional limiting factors:**

 (1) Cultural (participants from 16 countries, and from Europe, Asia and South America) differences may have possibly influenced the individuals’ understanding and interpretation of the musculoskeletal pain.

 (2) The gender-related differences in MSDs  of the participants was not analyzed in this meta-analyses.

 (3) Habitual physical activity of the participants was not analyzed in this study. All these limitations should be presented at the end of the Discussion.

**Reply: **Thank you for your thoughtful comments. We have now added the suggested limitations to the end of the Discussion section. Specifically, we have acknowledged the potential influence of cultural differences among participants from 16 countries across Europe, Asia, and South America on the perception and interpretation of musculoskeletal pain. We have also noted that gender-related differences in MSDs and habitual physical activity were not analyzed due to limitations in the available data. These additions help to more clearly frame the boundaries of the study’s findings.

 In conclusion, I recommend to accept this manuscript for indexing in bibliographic databases given that the authors address all the minor corrections.

**Specific Comments**

**Abstract**

 Page 1. Please add the information about age range and sex (% female) of the participants.

**Reply: **Thank you for your valuable suggestion. We have now included the age range of the participants (17–45 years) in both the Abstract and the Study Selection and Characteristics sections to enhance the readers’ understanding of the studied population. However, we were unable to report the sex distribution (% female) of the participants due to the limitations of the secondary data. Specifically, the primary studies included in this systematic review did not consistently report participants' sex, which precluded us from providing this information.

**Results**

 Page 5, Study selection characteristics. Please add information about age range and sex (%female) of the participants.

**Reply: **Thank you for your valuable suggestion. We have now included the age range of the participants (17–45 years) in both the Abstract and the Study Selection and Characteristics sections to enhance the readers’ understanding of the studied population. However, we were unable to report the sex distribution (% female) of the participants due to the limitations of the secondary data. Specifically, the primary studies included in this systematic review did not consistently report participants' sex, which precluded us from providing this information.

**Discussion**

 Page 12. Please add more limiting factors (see General Comments)

**Reply:** Thank you for your thoughtful comments. We have now added the suggested limitations to the end of the Discussion section. Specifically, we have acknowledged the potential influence of cultural differences among participants from 16 countries across Europe, Asia, and South America on the perception and interpretation of musculoskeletal pain. We have also noted that gender-related differences in MSDs and habitual physical activity were not analyzed due to limitations in the available data. These additions help to more clearly frame the boundaries of the study’s findings.

 Are the rationale for, and objectives of, the Systematic Review clearly stated?

 Yes

 Are sufficient details of the methods and analysis provided to allow replication by others?

 Yes

 Is the statistical analysis and its interpretation appropriate?

 Yes

 Are the conclusions drawn adequately supported by the results presented in the review?

 Yes

 If this is a Living Systematic Review, is the ‘living’ method appropriate and is the search schedule clearly defined and justified? (‘Living Systematic Review’ or a variation of this term should be included in the title.)

 Yes

# Reviewer report: Reviewer response for version 1

**DOI:** 10.5256/f1000research.167132.r321523

**Published:** 2024-09-12

**Authors:** Anna Zalewska

**Affiliations:** 1 Faculty of Health Sciences, University of Lomza, Lomza, Poland

Thank you for the opportunity to review this paper. This is a timely issue to explore. The aim of this study was to assess the prevalence of musculoskeletal symptoms in online students. The manuscript meets all the requirements for a systematic review. I have two minor comments for the authors.

 - Authors should also remember to exclude so-called grey literature from their research.

 - Research articles usually do not use the word „our” and regularly use passive verbs.

Are the rationale for, and objectives of, the Systematic Review clearly stated?

Yes

Is the statistical analysis and its interpretation appropriate?

I cannot comment. A qualified statistician is required.

If this is a Living Systematic Review, is the ‘living’ method appropriate and is the search schedule clearly defined and justified? (‘Living Systematic Review’ or a variation of this term should be included in the title.)

Yes

Are sufficient details of the methods and analysis provided to allow replication by others?

Yes

Are the conclusions drawn adequately supported by the results presented in the review?

Yes

Reviewer Expertise:

Health Sciences

I confirm that I have read this submission and believe that I have an appropriate level of expertise to confirm that it is of an acceptable scientific standard.
